# Evaluating Associations Between Ankylosing Spondylitis, Torque Teno Virus and Polymorphisms in Interleukin 6 and Vitamin D Receptor Genes

**DOI:** 10.1111/iji.70033

**Published:** 2026-01-12

**Authors:** Dănuț Cimponeriu, Lavinia‐Mariana Berca, Irina Radu, Mihai Toma, Remus Nica, Silvia Nica, Roxana Măciucă

**Affiliations:** ^1^ Department of Genetics Faculty of Biology University of Bucharest Bucharest Romania; ^2^ Molecular Biology Department National Research and Development Institute for Food Bioresources – IBA Bucharest Romania; ^3^ Faculty of Medicine Titu Maiorescu University Bucharest Romania; ^4^ Central Military Emergency University Hospital Dr. Carol Davila Bucharest Romania; ^5^ Carol Davila University of Medicine and Pharmacy Bucharest Romania

**Keywords:** ankylosing spondylitis, interleukin‐6, VDR, polymorphisms, TTV

## Abstract

The etiology of ankylosing spondylitis (AS) is complex and not yet fully understood. Interleukin‐6 (IL‐6), vitamin D and the vitamin D receptor (VDR) play an important role in modulating immune response, and Torque teno virus is considered a marker of immune status.

This case–control study aimed to investigate the predisposition to AS. A total of 85 patients with AS and 100 clinically healthy individuals were included. *VDR* polymorphisms (rs2228570, rs1544410, rs7975232, rs731236) were genotyped using the PCR‐RFLP technique, while for the *IL*‐6 ‐174 G>C (rs1800795) polymorphism the tetra‐primer ARMS‐PCR technique was used. The presence of TTV was detected using the hemi‐nested PCR technique.

Our findings indicate a statistically significant association between TTV and AS (*p* = 0.035). C allele of both rs1800795 polymorphism in main groups (*p* = 0.027) and rs731236 polymorphism in women subgroups (*p* = 0.036) may be linked to an increased susceptibility to AS. However, none of these associations reach statistical significance after Bonferroni correction. Furthermore, within female subgroups, a significant association was found between the T allele of rs1544410 polymorphism and AS (*p* = 0.000038, corrected *p* = 0.00076). A significant association was also observed between the TT genotype of rs2228570 polymorphism, TTV and AS (*p* = 0.029). Haplotype analysis revealed that certain VDR haplotypes may confer either a protective effect against AS or an increased risk of developing the condition. Notably, rs1544410 polymorphism or a linked polymorphism may influence AS susceptibility. In conclusion, our data suggest that TTV and *VDR* polymorphisms may be associated with an increased risk of developing AS, indicating that these markers could potentially be used in the future for earlier diagnosis and more targeted treatment of the disease.

## 1 Introduction

Ankylosing spondylitis (AS) is a chronic inflammatory disease with an onset that typically occurs before the age of 45 years (Boel et al. 2022). The aetiology and progression of AS are influenced by a combination of genetic and non‐genetic risk factors. HLA‐B27, the microbiome and viral or bacterial infections have a particular importance among these risk factors for AS (Brown et al. 2016; Babaie et al. 2018; Díaz‐Peña et al. 2020). Genome‐wide association studies (GWAS) have revealed multiple genes implicated in the aetiology of AS by analysing single‐nucleotide polymorphisms (SNPs) (Nancy et al. 2021).

Interleukin‐6 (IL‐6), a member of the IL‐6 cytokine family, is a protein with both pro‐inflammatory and anti‐inflammatory properties, playing a critical role in modulating the immune response (Kang et al. 2020). IL‐6 is essential for the differentiation of T cells into Th17 cells, which contribute to the pathology of spondyloarthritis, including AS (van den Berg et al. 2013; Xiong et al. 2022). The *IL6* gene, which encodes this protein, is located on chromosome 7 (7p15.3). It consists of 5 exons and 4 introns, and several polymorphisms have been identified within this gene that influence circulating IL‐6 levels, particularly those located in the promoter region. Among these, the most extensively studied polymorphism is rs1800795 (IL‐6 ‐174G >C), which has been associated with multiple conditions, including systemic lupus erythematosus (Liu et al. 2021), rheumatoid arthritis (Hao et al. 2024), psoriatic arthritis (Cubino et al. 2016), cervical cancer (Wagh et al. 2021), type 2 diabetes mellitus (Ayelign et al. 2021; Cheng et al. 2022).

Vitamin D is a molecule involved in regulating various processes, such as phosphate and calcium metabolism, bone mineral density and immune response (Gasperini et al. 2023). Deficiency in vitamin D has been investigated as a potential risk factor for developing autoimmune disorders (Murdaca et al. 2019) and has been linked to genetic variation in *VDR* gene (Daryabor et al. 2023). The vitamin D receptor (VDR), a nuclear receptor, has an important regulatory role by inhibiting lymphocyte responses mediated by Th1 and Th17 (Hwang et al. 2023). It also modulates the innate immune responses to various pathogens (Lagishetty et al. 2011). Several polymorphisms have been identified in the *VDR* gene (located on 12q13.11) with the most frequently studied being: rs2228570 (VDR FokI C>T; exon 2), rs1544410 (VDR BsmI C>T, intron 8), rs7975232 (VDR ApaI A>C, intron 8) and rs731236 (VDR TaqI C>T, exon 9). These polymorphisms are named after the restriction enzymes that detect them in RFLP assays. The BsmI, ApaI and TaqI affect VDR protein structure, whereas the FokI polymorphism influences transcriptional activation. Depending on the translation start site, this polymorphism leads to two distinct protein isoforms: The longer 427‐amino variant (associated with the T allele) and the shorter 424‐amino variant (associated with the C allele). These SNPs seems to be involved in the aetiology of multiple diseases (Bizzaro et al. 2017)—for example, FokI and ApaI in multiple sclerosis (AL‐Eitan and Darabseh 2025); TaqI and ApaI in type 1 diabetes mellitus (Kamel et al. 2014); FokI, ApaI (S. Zhang et al. 2025) and BsmI (Luo et al. 2012) in systemic lupus erythematosus.

Torque teno virus (TTV) is a non‐enveloped virus from the *Anelloviridae* family, with a genome consisting of a single strand of circular DNA (Nishizawa et al. 1997). This virus is widely distributed worldwide, found in 80%–90% of the human population (Cebriá‐Mendoza et al. 2023), and in 71.5% of the population in Romania (Spandole‐Dinu et al. 2018). TTV is considered a marker of immune status (Gore et al. 2023) and have been linked to certain autoimmune diseases (Sabbaghian et al. 2024) including multiple sclerosis and systemic lupus erythematosus (Borkosky et al. 2012; Costa et al. 2012), though its precise role in disease pathophysiology remains unclear.

This article aims to analyse the association between AS, SNPs in *IL6* gene (rs1800795) and *VDR* gene (rs2228570, rs1544410, rs7975232, rs731236), and the presence of TTV.

## 2 Materials and Methods

### 2.1 Selection of Subjects and Data Collection

This case–control study included 85 patients diagnosed with AS group and 100 clinically healthy individuals (HC group) who live in the metropolitan area of Bucharest, aged between 50 and 66 years. Patients and controls were unrelated and matched by gender and age. Patients attended visits at Central Military Emergency University Hospital Dr. Carol Davila between January 2021 and February 2023. They were included in the present study because they met the Modified New York Criteria, presenting radiologic evidence of definite sacroiliitis (sacroiliac joint x‐rays or MRI) and at least one of the specific clinical features of AS (low back pain and stiffness for more than 3 months, not improved with rest, chest expansion decreased and limited lumbar spinal motion). Healthy controls were selected from individuals who exhibited neither clinical manifestations of AS nor abnormal values in routine laboratory investigations.

This study has been approved by the Ethics Committee of National Research and Development Institute for Food Bioresources—IBA (approval number: 212/20.02.2021). All participants were informed about the purpose of the study and signed an informed consent form.

### 2.2 Methods and Protocols

DNA was extracted with Promega Wizard Genomic DNA Purification Kit, according to the manufacturer's protocol, from peripheral blood (300 µL) collected in EDTA anticoagulant tubes. *IL6* polymorphism (rs1800795) was genotyped using tetra‐primer ARMS‐PCR technique (Ye et al. 2001), while the genotyping of *VDR* polymorphisms was performed by PCR‐RFLP technique, based on different protocols previously described: rs2228570 (Minamitani et al. 1998), rs1544410 (Alexiu‐Toma et al. 2022), rs7975232 (Riggs et al. 1995) and rs731236 (Riggs et al. 1995). The presence of TTV was identified using a hemi‐nested PCR protocol (Ninomiya et al. 2007). Primers sequences and protocols for SNPs and TTV detection are presented in Table 1 and Table 2.

**TABLE 1:** Primers sequences for *IL6* and *VDR* SNPs.

Gene	SNP	Direction	Primer sequence	Primer location	Reference
*IL6*	rs1800795	Forward inner primer (G allele)	5′ GCACTTTTCCCCCTAGTTGTGTCTTCCG 3′	Promoter region	Ye et al. 2001
		Reverse inner primer (C allele)	5′ ATTGTGCAATGTGACGTCCTTTAGCTTG 3′	Promoter region	
		Forward outer primer	5′ GACTTCAGCTTTACTCTTTGTCAAGACA 3′	Promoter region	
		Reverse outer primer	5′ GAATGAGCCTCAGACATCTCCAGTCCTA 3′	Promoter region	
*VDR*	rs731236	Forward primer	5′CAGAGCATGGACAGGGAGCAA 3′	Intron 8	Riggs et al. 1995
		Reverse primer	5′GCAACTCCTCATGGCTGAGGTCTC 3 ‘	Exon 9	
	rs7975232	Forward primer	5′CAGAGCATGGACAGGGAGCAA3′	Intron 8	Riggs et al. 1995
		Reverse primer	5′GCAACTCCTCATGGCTGAGGTCTC3′	Exon 9	
	rs1544410	Forward primer	5′TCCAAAGTTTTGTACCCTGCC3′	Intron 8	Alexiu‐Toma et al. 2022
		Reverse primer	5′TTCGTAGGGGGGATTCT3′	Intron 8	
	rs2228570	Forward primer	5′AGCTGGCCCTGGCACTGACTCTGCTCT3′	Exon 2	Minamitami et al. 1998
		Reverse primer	5′ATGGAAACACCTTGCTTCTTCTCCCTC 3′	Exon 2	

**TABLE 2:** Primers sequences for Torque teno virus detection.

Primer	Direction	Primer sequence	Primer location
NG696	Forward primer	5′‐ATGGTTTCCTACAGTTGCATGG‐3′	nt 1766–1787
NG697	Reverse primer	5′‐CAGAGTACAATAGAGTCTGGCT‐3′	nt 562–583
NG698	Forward primer	5′‐TCTTACCTTCCTTCTGCGTCTG‐3′	nt 1852–1873
NG699	Reverse primer	5′‐TCTTACCTTCCTTCTGCGTCTG‐3′	nt 506–527
NG702	Forward primer	5′‐GGAGAGTTACAGGCCCTTGC‐3′	nt 2205–2224
NG701	Reverse primer	5′‐AACTGTTGGCAGGCAAAACCTC‐3′	nt 730–751
NG716	Forward primer	5′‐ACAGCCCTCCAAGAAATCAACC‐3	nt 2228–2249
NG703	Reverse primer	5′‐GGTGATCTGGGAGGTGGTGC‐3′	nt 702–721

*Note*: Primer sequences and protocol adopted from Ninomiya et al. 2007.

### 2.3 Statistical Analysis

Data analysis was performed using the Simple Interactive Statistical Analysis (SiSa) online platform and statistical software package MedCalc version 23.4.1. Categorical data were compared using Fisher's exact test or *χ* ^2^ test. Associations between variables were considered statistically significant when the *p*‐value was below 0.05. Given multiple comparisons, Bonferroni correction method was applied to correct *p*‐values. The strength of association between variables was evaluated using odds ratios (OR) and their corresponding 95% confidence intervals (CI). Hardy–Weinberg equilibrium was assessed for all SNPs using the *χ* ^2^ test. Haplotype diversity of VDR gene polymorphisms was estimated using the SHEsis online platform (Shi and He 2005; Li et al. 2009).

## 3 Results

### 3.1 Baseline Characteristics of the Subjects

Average age in the AS group was 58.91 ± 3.17 (52–65), while in the control group was 58.1 ± 3.49 (50–66). Of the 85 patients, 62.4 % (*n* = 53) were males and 37.6% (*n* = 32) were females; of the 100 healthy controls, 61% (*n* = 61) were males and 39% (*n* = 39) were females.

Analysis of subject characteristics revealed significant associations between AS and skin diseases, kidney disorders, bone fractures, family history of inflammatory or autoimmune diseases, history of AS and TTV presence. The characteristics of the studied subjects are presented in Table 3.

**TABLE 3:** Subjects’ clinical and paraclinical characteristics.

	AS group *n* = 85		HC group *n* = 100				
Variables	*n*	%	*n*	%	*p*‐value	Corrected *p*	OR (95% CI)
Average age (years) ± SD	58.91 ± 3.17 (52–65)		58.1 ± 3.49 (50–66)		NS	NS	
**Sex**							
Female	32	37.6	39	39			
Male	53	62.4	61	61	0.850	NS	0.94 (0.52–1.71)
**Coffee consumption**							
Yes	32	37.6	49	49			
No	53	62.5	51	51	0.121	NS	0.62 (0.34–1.13)
**Heart diseases** ^a^							
Yes	17	20	0	0			
No	68	80	0	0	NA	NA	
**Hypertension**							
Yes	23	27.1	0	0			
No	62	72.9	0	0	NA	NA	
**Type 2 diabetes mellitus**							
Yes	12	14.1	0	0			
No	73	85.9	0	0	NA	NA	
**Inflammatory bowel diseases** ^b^							
Yes	11	12.9	0	0			
No	74	87.1	0	0	NA	NA	
**Skin diseases** ^c^							
Yes	14	16.5	2	2			
No	71	83.5	98	98	**0.0005** **^d^**	**0.001**	9.66 (2.13–43.86)
**Urolithiasis/microalbuminuria**							
Yes	22	25.9	12	12			
No	63	74.1	88	88	**0.015** **^d^**	**0.015**	2.56 (1.18–5.55)
**Previous bone fractures**							
Yes	29	34.1	19	19			
No	56	65.9	81	81	**0.019** **^d^**	**0.019**	2.21 (1.13–4.32)
**Family history of ankylosing spondylitis**							
Yes	11	12.9	3	3			
No	74	87.1	97	97	**0.013** **^d^**	**0.026**	4.80 (1.29–17.85)
**Family history of other inflammatory or autoimmune diseases**							
Yes	26	30.6	13	13			
No	32	37.6	71	71	**0.0001** **^d^**, **^e^**	**0.0002**	4.44 (2.02–9.74)
Unknown	27	31.8	16	16			
**HLA‐B27**							
Yes	50	58.8	6	6			
No	21	24.7	5	5	0.30^e^	.30	1.98 (0.55–7.22)
Unknown	14	16.5	89	89			
**Torque teno virus**							
Yes	58	68.2	53	53			
No	27	31.8	47	47	**0.035** **^d^**	**0.035**	1.91 (1.04–3.48)

*Note*: Data are presented as numbers (*n*) and percentage (%)

Abbreviations: AS group = Ankylosing spondylitis group; Corrected *p* = *p* value after applying Bonferroni correction; HC group = Healthy controls group; CI = Confidence interval; OR = Odds ratio.

^a^ Myocardial infarction, coronary artery disease

^b^ Crohn disease, ulcerative colitis

^c^ psoriasis, vitiligo, atopic dermatitis

^d^ The significance was calculated by *χ* ^2^ test or Fisher exact test and two‐tailed *p* < 0.05 was considered significant.

^e^ The result was obtained by comparing participants with and without the variable; individuals who were unsure of their status were excluded from the analysis.

Following the gender distribution (Table 4), it was found that in the patient group comorbidities were more frequent in males that in females, particularly hypertension (60.9%), heart conditions (70.9%) and kidney conditions (59.1%) were recorded.

**TABLE 4:** Subjects’ clinical and paraclinical characteristics—subgroup analysis by gender.

	Women subgroup					Men subgroup				
	AS subgroup *n* = 32		HC subgroup *n* = 39		*p*‐value; OR (95% CI); corrected p	AS subgroup *n* = 53		HC subgroup *n* = 61		*p*‐value; OR (95% CI); corrected *p*
Variables	*n*	%	*N*	%		*n*	%	*n*	%	
**Coffee consumption**										
Yes	10	31.3	18	46.1		23	43.4	31	51.0	
No	22	68.7	21	53.9	0.203; 0.53 (0.02–1.41)	30	56.6	30	49.0	0.429; 0.742 (0.35–1.55)
**Heart diseases** ^a^										
Yes	5	15.6	0	0		12	22.6	0	0	
No	27	84.4	39	100	NA	41	77.4	61	100	NA
**Hypertension**										
Yes	9	28.1	0	0		14	26.4	0	0	
No	23	71.9	39	100	NA	39	73.6	61	100	NA
**Type 2 diabetes mellitus**										
Yes	5	15.6	0	0		7	13.2	0	0	
No	27	84.4	39	100	NA	46	86.8	61	100	NA
**Inflammatory bowel diseases** ^b^										
Yes	7	21.9	0	0		4	7.5	0	0	
No	25	78.1	39	100	NA	49	92.5	61	100	NA
**Skin diseases** ^c^										
Yes	5	15.6	0	0		9	17.0	2	3.3	
No	27	84.4	39	100	NA	44	83.0	59	96.7	**0.017**; 6.03 (1.24–29.24); 0**.034**
**Urolithiasis/microalbuminuria**										
Yes	9	28.1	3	7.7		13	24.5	9	14.8	
No	23	71.9	36	92.3	**0.028**; 4.70 (1.15–19.14)	40	75.5	52	85.2	0.279; 1.88 (0.73–4.83)
**Previous bone fractures**										
Yes	16	50.0	8	20.5		13	24.5	11	18.0	
No	16	50.0	31	79.5	**0.010**; 3.88 (1.37–10.98)	40	75.5	50	82.0	0.396; 1.48 (0.59–3.65)
**Family history of ankylosing spondylitis**										
Yes	5	15.6	3	7.7		6	11.3	1	1.6	
No	27	84.4	36	92.3	0.326; 2.22 (0.49 – 10.09); NS	47	88.7	60	98.4	**0.041**; 7.66 (0.89–65.77); NS
**Family history of other inflammatory or autoimmune diseases**										
Yes	11	34.3	6	15.4		15	28.3	8	13.1	
No	9	28.1	27	69.2	**0.008** **^e^**; 5.50 (1.58–19.17); 0.**0016**	23	43.4	44	72.1	0.**019** ^e^; 3.59 (1.32–9.71); 0.**038**
Unknown	12	37.8	6	15.4		15	28.3	9	14.8	
**HLA‐B27**										
Yes	22	68.8	3	7.7		28	52.8	3	4.9	
No	5	15.6	2	5.1	0.353**^e^**; 2.93 (0.38–22.39)	16	30.2	3	4.9	0.55^e^; 1.75 (0.32–5.55)
Unknown	5	15.6	34	87.2		9	17.0	55	90.2	
**Torque teno virus**										
Yes	23	71.9	20	51.2		35	66.0	33	54.1	
No	9	28.1	19	48.8	0.128; 2.428 (0.89–6.56)	18	34.0	28	45.9	0.194; 1.65 (0.77–3.53)

*Note*: Data are presented as numbers (*n*) and percentage (%)

Abbreviations: AS group = Ankylosing spondylitis group; CI = Confidence interval; Corrected *p* = *p* value after applying Bonferroni correction; HC group = Healthy controls group; OR = odds ratio.

^a^ Myocardial infarction, coronary artery disease

^b^ Crohn disease, ulcerative colitis

^c^ psoriasis, vitiligo, atopic dermatitis

^d^ The significance was calculated by *χ* ^2^ test or Fisher exact test and two‐tailed *p* < 0.05 was considered significant.

^e^ The result was obtained by comparing participants with and without the variable; individuals who were unsure of their status were excluded from the analysis.

Within the women subgroups, statistically significant differences were observed for bone fractures (16/16 vs. 8/31, OR 3.88; 95% CI 1.37–10.98; *p* = 0.01) and family history of inflammatory or autoimmune diseases (11/9 vs. 6/27; OR 5.5; 95% CI 1.58–19.17; *p* = 0.008, corrected *p* = 0.016).

Men diagnosed with AS were more likely to have a positive family history of inflammatory or autoimmune diseases than clinically healthy men (15/23 vs. 8/44; OR 3.59; 95% CI 1.32–9.71, *p* = 0.019, corrected *p* = 0.038).

Among patients, bone fractures were more frequently reported by women compared to men (50% vs. 24.52%; OR 3.08; 95% CI 1.21–7.83; *p* = 0.016) (Table 5).

**TABLE 5:** Gender‐based subgroup analysis of clinical and paraclinical characteristics of AS patients

	AS women subgroup (*n* = 32)		AS men subgroup (*n* = 53)			
Variable	n	%	n	%	*p*‐value	OR (95% CI)
**Coffee consumption**						
Yes	10	31.3	23	43.4		
No	22	68.7	30	56.6	0.266	0.593 (0.24–1.49)
**Heart diseases** ^a^						
Yes	5	15.6	12	22.6		
No	27	84.4	41	77.4		
**Hypertension**						
Yes	9	28.1	14	26.4	0.006	1.09 (0.48–2.91)
No	23	71.9	39	73.6		
**Type 2 diabetes mellitus**						
Yes	5	15.6	7	13.2	0.757	1.22 (0.35–4.22)
No	27	84.4	46	86.8		
**Inflammatory bowel diseases** ^b^						
Yes	7	21.9	4	7.5		
No	25	78.1	49	92.5	0.073	3.43 (0.92–12.85)
**Skin diseases** ^c^						
Yes	5	15.6	9	17.0		
No	27	84.4	44	83.0	0.765	0.91 (0.27–2.99)
**Urolithiasis/microalbuminuria**						
Yes	9	28.1	13	24.5	0.012	1.20 (0.45–3.25)
No	23	71.9	40	75.5		
**Previous bone fractures**						
Yes	16	50.0	13	24.5		
No	16	50.0	40	75.5	**0.016** **^d^**	3.08 (1.21–7.83)
**Family history of ankylosing spondylitis**						
Yes	5	15.6	6	11.3		
No	27	84.4	47	88.7	0.579	1.45 (0.40–5.21)
**Family history of other inflammatory or autoimmune diseases**						
Yes	11	34.3	15	28.3		
No	9	28.1	23	43.4	0.727**^e^**	1.87 (0.63 –5.60)
Unknown	12	37.8	15	28.3		
**HLA‐B27**						
Yes	22	68.8	28	52.8		
No	5	15.6	16	30.2	0.118**^e^**	2.51 (0.80–7.93)
Unknown	5	15.6	9	17.0		
**Torque teno virus**						
Yes	23	71.9	35	66.0		
No	9	28.1	18	34.0	0.749	1.31 (0.50–3.42)

*Note*: Data are presented as numbers (*n*) and percentage (%)

Abbreviations: AS group = Ankylosing spondylitis group; CI = Confidence interval; HC group = Healthy controls group; OR = Odds ratio.

^a^ Myocardial infarction, coronary artery disease

^b^ Crohn disease, ulcerative colitis

^c^ psoriasis, vitiligo, atopic dermatitis

^d^ The significance was calculated by *χ* ^2^ test or Fisher exact test and two‐tailed *p* < 0.05 was considered significant.

^e^ The result was obtained by comparing participants with and without the variable; individuals who were unsure of their status were excluded from the analysis.

Regarding the presence of HLA‐B27 marker, 16.5% of patients were not tested. Of the 83.5% tested, 70.4% presented the HLA‐B27 marker, and 29.6% had a negative result. The marker was present at a higher percentage in men (56%) than in women (44%). In the control group, only 11% of subjects were tested, and 54.5% had a positive result; among the positives, 50% were women and 50% were men. The HLA‐B27 marker was also identified in 72.7% of patients diagnosed with an inflammatory bowel disease, of which 75% were women and 25% were men, as well as in 65.4% of patients with a family history of inflammatory and autoimmune diseases. No significant associations were found between HLA‐B27, TTV and any of the genetic polymorphisms in the *IL6* and *VDR* genes investigated in the present study.

### 3.2 TTV Presence

Patients had a higher TTV positivity rate compared to controls (68.2% vs. 53%, *p* = 0.035) (Table 3). Among patients, TTV was more prevalent in males than in females (60.3% vs. 39.6%) (Table 5). However, when stratified by gender, the difference did not reach statistical significance. In addition, TTV infection combined with a family history of inflammatory or autoimmune diseases was more commonly observed in AS group than in the HC group (16/42 vs. 7/77; OR 4.19; 95% CI: 1.6–10.99; *p* = 0.003).

### 3.3 SNPs Distribution

According to the Hardy–Weinberg law, there were no differences between the observed and expected genotype distributions for the SNPs located in the *IL6* and *VDR* genes in either the patient or control groups. The distribution of genotypes and alleles is presented in Table 6.

**TABLE 6:** Distribution of genotypes and alleles in ankylosing spondylitis patients and controls.

		Ankylosing spondylitis *n* = 85		Control *n* = 100				
Statistic model	Genotype/Allele	*n*	Frequency	*n*	Frequency	*p*‐value	Corrected *p*	OR (95% CI)
**SNPs**								
**rs1800795**								
	GG	28	0.329	49	0.490	0.086		
	GC	44	0.518	40	0.400			
	CC	13	0.153	11	0.110			
	G‐allele	100	0.588	138	0.690			
	C‐allele	70	0.412	62	0.310			
Dominant	G (GG+GC vs. CC)	72 /13		89/11		0.386		0.69 (0.29–1.62)
	C (CC+GC vs. GG)	57/28		51/49		**0.027** **^a^**	**0.054**	1.96 (1.08–3.56)
Recessive	G (GG vs. GC+CC)	28/57		49/51		**0.027** **^a^**	**0.054**	0.51 (0.28–0.93)
	C (CC vs. GC+GG)	13/72		11/89		0.386		1.46 (0.62–3.46)
**rs731236**								
	TT	28	0.329	39	0.390	0.637		
	TC	46	0.541	51	0.510			
	CC	11	0.130	10	0.100			
	T‐allele	102	0.600	129	0.645			
	C‐allele	68	0.400	71	0.355			
Dominant	T (TT+TC vs. CC)	74/11		90/10		0.529		0.75 (0.30–1.86)
	C (CC+TC vs. TT)	57/28		61/39		0.393		1.30 (0.71–2.38)
Recessive	T (TT vs. CC+TC)	28/57		39/61		0.393		0.77 (0.42–1.41)
	C (CC vs. TT+TC)	11/74		10/90		0.529		0.62 (0.54–3.32)
**rs7975232**								
	AA	21	0.247	28	0.280	0.678		
	AC	48	0.568	50	0.500			
	CC	16	0.185	22	0.220			
	A‐allele	90	0.529	106	0.530			
	C‐allele	80	0.471	94	0.470			
Dominant	A (AA+AC vs. CC)	69/16		78/22		0.594		1.22 (.59–2.50)
	C (CC+AC vs. AA)	64/21		72/28		0.612		1.19 (0.61–2.29)
Recessive	A (AA vs. CC+AC)	21/64		28/72		0.612		0.84 (0.44–1.63)
	C (CC vs. AA+AC)	16/69		22/78		0.594		0.82 (0.4–1.69)
**rs1544410**								
	CC	20	0.235	34	0.340	0.286		
	CT	50	0.588	52	0.520			
	TT	15	0.177	14	0.140			
	C‐allele	90	0.529	120	0.600			
	T‐allele	80	0.471	80	0.400			
Dominant	C (CC+CT vs. TT)	70/15		86/14		0.497		0.76 (0.34–1.68)
	T (AA+GA vs. CC)	65/20		66/34		0.118		1.67 (0.87–3.21)
Recessive	C (CC vs. TT+CT)	20/65		34/66		0.118		0.59 (0.31–1.14)
	T (TT vs. CC+CT)	15/70		14/86		0.497		1.31 (0.60–2.91)
**rs2228570**								
	CC	22	0.259	33	0.330	0.347		
	CT	36	0.424	44	0.440			
	TT	27	0.317	23	0.230			
	C‐allele	80	0.470	110	0.550			
	T‐allele	90	0.530	90	0.450			
Dominant	C (CC+TC vs. TT)	58/27		77/23		0.181		0.64 (0.33–1.23)
	T (TT+TC vs. CC)	63/22		67/33		0.291		1.41 (0.74–2.67)
Recessive	C (CC vs. TT+TC)	22/63		33/67		0.291		0.71 (0.37–1.34)
	T (TT vs. CC+TC)	27/58		23/77		0.181		1.56 (0.81–2.99)

*Note*: Data are presented as numbers (*n*) and frequency.

Abbreviations: CI = Confidence interval; Corrected *p* = *p* value after applying Bonferroni correction; OR = Odds ratio; SNP = Single nucleotide polymorphisms.

^a^ The significance was calculated by *χ* ^2^ test or Fisher exact test and two‐tailed *p* < 0.05 was considered significant.

When comparing the two main groups (AS and HC), we found that C allele of rs1800795 increased risk of AS, but the association was not statistically significant after Bonferroni correction (OR 1.96; 95% CI: 1.08–3.56; *p* = 0.027, corrected *p* = 0.054) (Table 6). The CC genotype was more frequent in the patient group than in the control group (15.3% vs. 11%), while the GG genotype was more frequent in the control group than in the patient group (49% vs. 32.9%). When distribution was stratified by gender, no significant differences between the two groups were observed for either males or females. Nevertheless, the CC genotype was more frequent identified among men than women (76.9% vs. 23.1%).

No significant associations were found for any of the *VDR* polymorphisms and AS when comparing the AS and HC groups. However, gender‐stratified analysis revealed significant association between AS and T allele of rs1544410 in the female subgroup (OR 9.64; 95% CI: 3.03–30.67; *p* = 0.000038; corrected *p* = 0.000076) (Table 7). Initially, C allele of rs731236 polymorphism was also associated with AS, but the difference did not remain statistically significant after Bonferroni correction (OR 3.39; 95% CI: 1.19–9.67; *p* = 0.036; corrected *p* = 0.072)

**TABLE 7:** Distribution of genotypes and alleles in women subgroup.

Statistic model	Genotype	AS group *n* = 32	HC group *n* = 39	*p*‐value	Corrected *p*	OR (95% CI)
	**rs7975232**					
	TT	7	19	0.003		
	TC	21	19			
	CC	4	1			
Dominant	T (TT+TC vs. CC)	28/4	38/1	0.139		0.18 (0.019–1.74)
	C (CC+TC vs. TT)	25/7	20/19	**0.036** **^a^**	**0.072**	3.39 (1.19–9.67)
Recessive	T (TT vs. CC+TC)	7/25	19/20	**0.036** **^a^**	**0.072**	0.29 (0.10–0.84)
	C (CC vs. TT+TC)	4/28	1/38	0.139		5.43 (0.58–51.225)
	**rs1544410**					
	CC	5	25	.000014		
	CT	22	13			
	TT	5	1			
Dominant	C (CC+CT vs. TT)	27/5	38/1	0.067		0.14 (0.02–1.29)
	T (TT+CT vs. CC)	27/5	14/25	**0.000038** **^a^**	**0.000076**	9.64 (3.03–30.67)
Recessive	C (CC vs. TT+CT)	5/27	25/14	**0.000038** **^a^**	**0.000076**	0.10 (0.0326–0.0329)
	T (TT vs. CT+CC)	5/27	1/38	0.067		7.03 (0.78–63.70)

Abbreviations: CI = Confidence interval; corrected *p* = *p* value after applying Bonferroni correction; OR = Odds ratio; SNP = Single nucleotide polymorphisms.

^a^ The significance was calculated by *χ* ^2^ test or Fisher exact test and two‐tailed *p* < 0.05 was considered significant.

In the female subgroup, the CC genotype of rs731236 was more frequent in patients than in controls (12.5% vs. 2.56%), whereas the TT genotype was more frequent in controls than in patients (48.72% vs. 21.88%). Similar results were observed for rs1544410, with the TT genotype being more prevalent among patients than controls (15.63% vs. 2.56%), while the CC genotype was more frequent in controls than in patients (64.10% vs. 15.63%).

The estimated VDR haplotypes were significantly associated with AS (Table 8). Some haplotypes were less common in the AS group, indicating a protective role against developing the condition (C‐C‐A‐T; C‐C‐A; C‐A‐T). On contrary, other haplotypes (T‐T‐A) were more frequent in the AS group, therefore carrying this haplotype increases the risk of AS.

**TABLE 8:** Haplotype analysis of the *VDR* SNPs.

Variable	Ankylosing spondylitis *n* = 85	Control *n* = 100	*p*‐value	OR (95% CI)
**rs2228570 ‐ rs1544410 ‐ rs7975232 ‐ rs731236**				
C C A C	0.00(0.000)	1.06(0.005)	0.341821	—
**C C A T**	**5.19 (0.031)**	**15.97 (0.080)**	**0.041756** **^a^**	**0.363 (0.132–0.999)**
C C C C	2.35(0.014)	0.00(0.000)	0.149189	1403.819 (69.418–28389.027)
C C C T	32.44(0.191)	44.78(0.224)	0.435424	0.817 (0.492–1.357)
C T A C	29.45(0.173)	36.89(0.184)	0.778879	0.926 (0.543–1.581)
C T A T	7.28(0.043)	6.44(0.032)	0.589596	1.345 (0.456–3.965)
C T C C	1.04(0.006)	1.24(0.006)	0.990328	0.984 (0.072–13.429)
C T C T	2.25(0.013)	3.62(0.018)	0.709740	0.728(0.136–3.891)
T C A T	9.33(0.055)	15.14(0.076)	0.421795	0.709 (0.305–1.647)
T C C T	40.68(0.239)	43.06(0.215)	0.582122	1.147 (0.704–1.867)
T T A C	33.93(0.200)	30.50(0.153)	0.234298	1.385 (0.808–2.374)
**T T A T**	**4.83(0.028)**	**0.00(0.000)**	**0.026586** **^a^**	**1272.503 (70.433–22990.160)**
T T C C	1.22(0.007)	1.30(0.006)	0.936278	1.106 (0.093–13.198)
T C C C	0.01(0.000)	0.00(0.000)	0.908535	—
**rs2228570 ‐ rs1544410 ‐ rs7975232**				
**C C A**	**5.18(0.030)**	**17.10(0.085)**	**0.026645** **^a^**	**0.336 (0.123–0.919)**
C C C	35.52(0.209)	44.64(0.223)	0.741398	0.920 (0.559–1.513)
C T A	36.28(0.213)	43.69(0.218)	0.907709	0.971 (0.590–1.597)
C T C	3.02(0.018)	4.57(0.023)	0.732277	0.775 (0.179–3.360)
T C A	9.32(0.055)	15.12(0.076)	0.422675	0.709 (0.305–1.648)
T C C	39.98(0.235)	43.14(0.216)	0.652425	1.119 (0.686–1.826)
**T T A**	**39.22(0.231)**	**30.09(0.150)**	**0.048097** **^a^**	**1.697 (1.001–2.875)**
T T C	1.47(0.009)	1.65(0.008)		—
**rs1544410 ‐ rs7975232 ‐ rs731236**				
C A C	0.00(0.000)	1.05(0.005)		—
**C A T**	**14.57(0.086)**	**31.11(0.156)**	**0.040027** **^a^**	**0.506 (0.262–0.978)**
C C T	73.17(0.430)	87.84(0.439)	0.830175	0.956 (0.632–1.444)
T A C	63.37(0.373)	67.41(0.337)	0.497104	1.160 (0.756–1.778)
T A T	12.05(0.071)	6.42(0.032)	0.090090	2.287 (0.858–6.095)
T C C	2.37(0.014)	2.54(0.013)	0.920479	1.095 (0.184–6.512)
T C T	2.20(0.013)	3.63(0.018)	0.686297	0.708 (0.131–3.821)
C C C	2.26(0.013)	0.00(0.000)	0.102904	—
**rs2228570 ‐ rs1544410**				
C C	39.55(0.233)	61.54(0.308)	0.106323	0.682 (0.428–1.086)
C T	40.45(0.238)	48.46(0.242)	0.922392	0.976 (0.605–1.576)
T C	50.45(0.297)	58.46(0.292)	0.924855	1.022 (0.653–1.600)
T T	39.55(0.233)	31.54(0.158)	0.068296	1.619 (0.962–2.725)
**rs1544410 ‐ rs7975232**				
**C A**	**14.50(0.085)**	**32.20(0.161)**	**0.028906** **^a^**	**0.486 (0.252–0.938)**
C C	75.50(0.444)	87.81(0.439)	0.921472	1.021 (0.676–1.541)
T A	75.50(0.444)	73.80(0.369)	0.142231	1.366 (0.900–2.073)
T C	4.50(0.026)	6.19(0.031)	0.796041	0.850 (0.248–2.914)
**rs7975232 ‐ rs731236**				
A C	63.00(0.371)	68.42(0.342)	0.568298	1.132 (0.739–1.735)
A T	27.00(0.159)	37.58(0.188)	0.462810	0.816 (0.474–1.405)
C C	5.00(0.029)	2.58(0.013)	0.263825	2.318 (0.509–10.562)
C T	75.00(0.441)	91.42(0.457)	0.758936	0.938 (0.622–1.415)

*Note*: Data are presented as numbers (*n*) and frequency.

Abbreviations: CI = confidence interval, OR = odds ratio, SNP = single nucleotide polymorphisms.

^a^ The significance was calculated by *χ* ^2^ test or Fisher exact test and two‐tailed *p* < 0.05 was considered significant.

No significant differences were observed for rs228570 polymorphism between the patient and control groups; however, the situation changed when TTV presence was considered, and a significant association between AS and the TT genotype emerged. Therefore, carriers of rs2228570 TT genotype who tested positive for TTV were more frequently observed in the AS group than in the HC group (23.52% vs. 11%; OR 2.49; 95% CI 1.12–5.55, *p* = 0.029). However, gender‐stratified analysis did not reveal any significant differences.

## 4 Discussion

AS is a multifactorial disease with a partially understood aetiology, therefore it is necessary to study additional potential risk factors. In the present study, in the group of AS patients, statistically significant differences were observed for skin disease (*p* = 0.0005, corrected *p* = 0.0010), kidney diseases (*p* = 0.015), family history of inflammatory or autoimmune diseases (*p* = 0.0001, corrected *p* = 0.0002), family history of AS (*p* = 0.013, corrected *p* = 0.026) and bone fractures (*p* = .019). These findings are consistent with previously published results (Rodrigues et al. 2023; Kandregula et al. 2021; Meier et al. 2020; Hanson and Brown 2017).

IL‐6 is involved in modulating the immune response and maintaining homeostasis and serves as a marker of inflammation (Kaur et al. 2020). Therefore, a high level of IL‐6 in patients' serum has been correlated with increased disease severity in various conditions, including AS (Zhao et al. 2024; Du et al. 2022). Serum IL‐6 levels may be influenced by the presence of the rs1800795 polymorphism located in the promoter region of the gene. Previous studies have shown that patients with schizophrenia (Zakharyan et al. 2012) or diabetes mellitus (Ayelign et al. 2021) who carry the rs1800795 C allele have higher serum IL‐6 levels. In addition, the rs1800795 CC genotype has been linked to higher bone mineral density at the femoral neck and distal epiphysis of the radius bone in elderly Caucasian women (Ni et al. 2014). Initially, our data indicated a significant association between rs1800795 and AS; however, this association did not remain significant after Bonferroni correction. Nevertheless, a positive trend was observed between the C allele and AS risk (OR 1.96; 95% CI: 1.08–3.56; *p* = 0.027, corrected *p* = 0.054), suggesting that studies with larger sample sizes are needed to confirm this potential association.

Vitamin D deficiency has been linked to an elevated risk of inflammatory and autoimmune disorders, as vitamin D plays a key role in maintaining homeostasis and regulating the immune response to prevent excessive inflammation. Its pleiotropic effects include stimulating the synthesis of anti‐inflammatory cytokines (IL‐4, IL‐10) (Sloka et al. 2011) while suppressing the production of pro‐inflammatory cytokines (IL‐2, IL‐6, IL‐12, IL‐17, IL‐22) (Syed Khaja et al. 2024; Daryabor et al. 2023). Consequently, low vitamin D levels are associated with elevated IL‐6 production, which may contribute to the progression of inflammatory diseases (Wen et al. 2019). Various immune cells, including macrophages, dendritic cells, monocytes and activated T and B cells, express VDRs and contain enzymes (e.g., CYP27B1) that convert vitamin D into its biologically active form (Kamen and Tangpricha 2010).

In addition, polymorphisms in the *VDR* gene may alter the receptor's binding affinity for vitamin D, thereby reducing its ability to downregulate IL‐6 expression and mitigate the inflammatory cascade (Usategui‐Martín et al. 2022).

Another important role of vitamin D is suppressing Th17 cell synthesis, while promoting the differentiation of CD4^+^ T cells into Th2 and Treg cells (Syed Khaja et al. 2024). The expansion of the Th17 cell population plays a pivotal role in the pathogenesis of AS, a process partly mediated by IL‐6, which promotes the differentiation of T cells into Th17 cells (van den Berg et al. 2013).

Patients with AS, as well as those with other autoimmune diseases, generally have lower vitamin D levels than clinically healthy subjects (Ben‐Shabat et al. 2020); however, causality in this context remains unclear.

In a case–control study conducted on a sample of the Brazilian population (Neves et al. 2020), associations were observed between the polymorphisms rs7975232 and rs2228570, vitamin D deficiency, sex and the occurrence of AS or psoriatic arthritis. In the AS group, men carrying the rs7975232 AA genotype and the rs2228570 TT genotype were at higher risk of developing AS. Conversely, the rs7975232 CC genotype was identified as a protective factor for women diagnosed with psoriatic arthritis.

Bone fractures were reported more frequently in AS patients than in healthy controls, particularly among women with AS (Tables 3, 4, and 5). This difference may be partially explained by the mineral imbalance in the bones due to hormonal profiles, disease progression, vitamin D deficiency and age of the subjects. Deficiency in vitamin D may contribute to inflammation and reduce bone mineral density in AS patients (Neves et al. 2020). The female patients included in the present study were postmenopausal (mean age 58.59 ± 2.905 years) and therefore at increased risk of developing osteoporosis. Moreover, the development of postmenopausal osteoporosis might be substantially influenced by the dysfunction of the *VDR* gene. An association between VDR polymorphisms (ApaI rs7975232, BsmI rs1544410, TaqI rs731236) and osteoporosis was found in postmenopausal women from Belarus and Lithuania (Marozik et al. 2018). The rs1544410 CC genotype was approximately three times more prevalent in patients with osteoporosis than in controls. These findings may provide a potential explanation for the observed associations between the rs731236 and rs1544410 polymorphisms, bone fractures and AS in the female subgroups. Associations between rs731236 or rs1544410 and AS were reported previously (Cai et al. 2016), while other studies did not identify any statistically significant associations (P. Zhang et al. 2017).

Some of the estimated VDR haplotypes were linked to AS (Table 8). These findings suggest that the rs1544410, or a linked polymorphism, may contribute to AS susceptibility, confirming partially previous results (Marozik et al. 2018).

TTV have been linked to several diseases in the literature (Gore et al. 2023; Borkosky et al. 2012; Costa et al. 2012), but no association with AS has been reported to date. In our study, TTV was more frequently detected in AS patients than in healthy controls (68.2% vs. 53%, *p* = 0.035), although these values are lower than previously reported for the Romanian population (71.5%) (Spandole‐Dinu et al. 2018). In addition, TTV‐positive subjects carrying the rs2228570 TT genotype may be at increased risk for developing AS, underlying a potential interplay between genetic and non‐genetic factors in the aetiology of AS. Vitamin D provides antiviral protection by upregulating antimicrobial peptides with direct antiviral activity (White 2022), suggesting a potential link between low vitamin D levels and increased susceptibility to viral infections, such as TTV.

## 5 Conclusion

The data obtained in this study indicated that rs731236, rs1544410, rs1800795, rs2228570 and TTV may contribute to an increased risk of AS. These relations may be influenced by the gender of subjects. Specifically, rs1544410 was associated with AS only in the female subgroups. Moreover, the presence of the rs2228570 TT genotype in subjects with TTV may be associated with an increased susceptibility to AS. All these correlations may indicate a role in the pathophysiology of the disease through interactions between genetic and non‐genetic factors. Our study contributes to a better understanding of the AS aetiology and may facilitate the discovery of novel biomarkers for earlier diagnosis and more targeted treatment.

This study, however, presents several limitations that should be considered when interpreting the results. First, the relatively small sample size may have prevented the detection of weaker associations between the analysed variables and AS. Second, all patients were recruited from a single hospital within one geographical area (the metropolitan area of Bucharest), which limits the extrapolation of the findings, as they may be specific to that population. Third, considering that AS is a multifactorial disease, the present study assessed only a limited number of environmental factors, which does not provide a comprehensive perspective on their involvement in the disease aetiology. Therefore, larger studies including participants from multiple geographic regions and a broader range of environmental factors are needed to validate these findings.

## Funding

This study was supported by the Ministry of Research and Education, grant number PN 23 01 03 03.

## Consent

Informed consent was obtained from all individual participants included in the study.

## Conflicts of Interest

The authors declare no conflicts of interest.

## Data Availability

The data that support the findings in this study are available from the corresponding author by request.
